# Restoring Blood Pressure in Hypertensive Mice Fails to Fully Reverse Vascular Stiffness

**DOI:** 10.3389/fphys.2020.00824

**Published:** 2020-07-21

**Authors:** Jochen Steppan, Sandeep Jandu, William Savage, Huilei Wang, Sara Kang, Roshini Narayanan, Daniel Nyhan, Lakshmi Santhanam

**Affiliations:** ^1^Department of Anesthesiology and Critical Care Medicine, Johns Hopkins University, Baltimore, MD, United States; ^2^Department of Chemical and Biomolecular Engineering, Johns Hopkins University, Baltimore, MD, United States; ^3^Department of Biomedical Engineering, Johns Hopkins University, Baltimore, MD, United States

**Keywords:** hypertension, vascular stiffness, pulse wave velocity, reversal, vascular smooth muscle cells

## Abstract

**Background:**

Hypertension is a well-established driver of vascular remodeling and stiffening. The goal of this study was to evaluate whether restoring normal blood pressure (BP) fully restores vascular stiffness toward that of normotensive controls.

**Methods:**

C57Bl6/J male mice received angiotensin II (angII; 1 μg/kg/min) via infusion pump for 8 weeks (hypertension group: HH), angII for 4 weeks (hypertension group: H4), angII for 4 weeks followed by 4 weeks of recovery (reversal group: HN), or sham treatment (normotensive group: NN). BP, heart rate, and pulse wave velocity (PWV) were measured longitudinally. At the end of the study period, aortas were harvested for testing of vasoreactivity, passive mechanical properties, and vessel structure.

**Results:**

The HH group exhibited a sustained increase in BP and PWV over the 8-week period (*p* < 0.01). In the HN group, BP and PWV increased during the 4-week angII infusion, and, though BP was restored during the 4-week recovery, PWV exhibited only partial restoration (*p <* 0.05). Heart rate was similar in all cohorts. Compared to NN controls, both HH and HN groups had significantly increased wall thickness (*p <* 0.05 HH vs. NN, *p <* 0.01 HN vs. NN), mucosal extracellular matrix accumulation (*p <* 0.0001 HH vs. NN, *p <* 0.05 HN vs. NN), and intralamellar distance (*p <* 0.001 HH vs. NN, *p <* 0.01 HN vs. NN). Both intact and decellularized vessels were noted to have significantly higher passive stiffness in the HH and H4 cohorts than in NN controls (*p <* 0.0001). However, in the HN cohort, intact vessels were only modestly stiffer than those of NN controls, and decellularized HN vessels were identical to those from the NN controls. Compared to NN controls, the HH and HN cohorts exhibited significantly diminished phenylephrine-induced contraction (*p <* 0.0001) and endothelium-dependent vasodilation (*p <* 0.05).

**Conclusion:**

Hypertension causes a significant increase in *in vivo* aortic stiffness that is only partially reversible after BP normalization. Although hypertension does lead to matrix stiffening, restoration of BP restores matrix mechanics to levels similar to those of normotensive controls. Nevertheless, endothelial and vascular smooth muscle cell dysfunction persist after restoration of normotension. This dysfunction is, in part, responsible for augmented PWV after restoration of BP.

## Introduction

Hypertension is a multifactorial disease that is associated with a multitude of comorbidities, such as vascular stiffness (Franklin and Levy, 2011; Steppan et al., 2011; AlGhatrif et al., 2013; Verwoert et al., 2014). Indeed, hypertension and vascular stiffness are interdependent, with hypertension leading to vascular stiffening and vice versa (Mitchell, 2014). Prior studies have shown a clear increase in pulse wave velocity (PWV), a well-accepted index of *in vivo* vascular stiffness, with elevated blood pressure (BP) and associated endothelial dysfunction (O’Rourke and Mancia, 1999; McEniery et al., 2010; AlGhatrif et al., 2013). Physiologically, mechanical forces that regulate endothelial cells include hoop stress imposed by the mean arterial pressure (MAP) and shear stress due to blood flow (Humphrey et al., 2015). The latter in particular is a key regulator of endothelial nitric oxide synthase-dependent nitric oxide production and endothelial function and thence, vascular tone and smooth muscle cell (SMC) function (Davis et al., 2001; Boo et al., 2002a, b; Verwoert et al., 2014). The vascular matrix and the vascular SMCs (VSMCs) are the main load-bearing elements in the vessel wall, and subtle changes in either can result in a remarkable increase in overall vascular stiffness. This stiffening of the vasculature leads to higher systolic and lower diastolic BP, which increases shear stress and can hasten the development of atherosclerosis. It can also cause degradation of the vessel wall and extracellular matrix (Hansson and Libby, 2006; Poels et al., 2012; Gimbrone and Garcia-Cardena, 2013). Recent studies have established the central role of VSMC tone and stiffness in overall vascular stiffness and shown that stiffer VSMCs can compensate for a compliant vascular matrix to maintain physiologically acceptable ranges of vascular stiffness (Sehgel et al., 2015; Steppan et al., 2017, 2019; Lacolley et al., 2018). However, the specific contributions of vascular matrix remodeling and VSMC dysregulation to overall stiffening remain less clear. The question is further complicated by the nuanced bidirectional communication between local matrix mechanics and resident VSMCs. These cell–matrix interactions are central to vasoreactivity and VSMC proliferation/motility, which are key determinants of the overall composition, structure, and function of the vessel (Moiseeva, 2001). It has been shown previously that increased VSMC strain can lead to phenotypic switching from a contractile to a synthetic phenotype, which results in arterial fibrosis and decreased arterial compliance (Qu et al., 2007; Tosun and McFetridge, 2015). However, few studies have addressed whether lowering BP in hypertensive individuals will ameliorate vascular stiffening or even restore it to normotensive levels (Stewart et al., 2006). Understanding whether vascular stiffening can be reversed is of particular importance given that patient adherence to prescribed BP medications remains low and almost half of patients with hypertension have insufficient control of their BP (Mozaffarian et al., 2015). The goal of this study was to evaluate whether re-establishing normal BP will recover vascular stiffness to levels in healthy controls and whether the VSMC or vascular matrix is the primary driver of stiffening.

## Materials and Methods

### Animals

C57Bl6/J male mice were used in this study and maintained in the Johns Hopkins University School of Medicine animal care facility. Mice were fed and watered *ad libitum* and maintained on a 12 h:12 h light-dark cycle. All procedures involving animals were approved by the Institutional Animal Care and Use Committee of the Johns Hopkins University. Mice were randomized to receive (1) angiotensin II (angII; at 1 μg/kg/min) via osmotic infusion pump (Alzet, Model 1004) for 8 weeks (HH group), (2) angII for 4 weeks (H4 group), (3) ang II for 4 weeks followed by 4 weeks of recovery (HN group), or (4) sham treatment (NN control group). BP, PWV (an index of arterial stiffness), and heart rate were measured longitudinally. Mice were sacrificed after 8 weeks, and aortas were harvested for testing of active (vasoreactivity) and passive mechanical properties (stress strain-relationship). All mice were 8–10 weeks old at the initiation of the study.

### Wire Myography

Vasoconstriction and vasorelaxation were examined by wire myography as previously described (Spiers and Padmanabhan, 2005; Steppan et al., 2017; Sun et al., 2018). Briefly, the thoracic aorta was excised from the surrounding soft tissues, cleaned, and then cut into 2 mm rings. Each ring was placed in Krebs solution [containing (in mmol/L) 118.3 NaCl, 4.7 KCl, 1.6 CaCl_2_, 1.2 KH_2_PO_4_, 25 NaHCO_3_, 1.2 MgSO_4_, and 11.1 dextrose at a pH of 7.4] and then transferred to a myograph chamber (DMT, Denmark) continuously bubbled with 95% O_2_ and 5% CO_2_ (37°C). The rings were stretched in 100 mg increments to a final tension of 600 mg. After the rings underwent passive stretching, we added KCl (60 mmol/L) to determine the viability of the vascular preparation and to obtain maximal contraction. Concentration–response curves were constructed for phenylephrine (10^–9^–10^–5^ mol/L). Next, we studied endothelial-mediated vasorelaxation by adding increasing doses of acetylcholine (10^–9^–10^–5^ mol/L) to vessels pre-constricted with phenylephrine (5 × 10^–6^ mol/L). Finally, we examined endothelial-independent vasorelaxation mediated by increasing doses of sodium nitroprusside (10^–9^–10^–5^ mol/L) in vessels pre-constricted with phenylephrine (5 × 10^–6^ mol/L).

### Tensile Testing

Tensile properties were measured in isolated intact and decellularized aortic rings as described previously (Jung et al., 2013; Steppan et al., 2014, 2019). Briefly, the thoracic aorta was excised from the surrounding soft tissues, cleaned, and then cut into 2 mm rings. Half of the rings were used intact and the other half decellularized by end-over-end shaking in 50 mM NH_4_OH + 0.2% SDS for 3 h, followed by three 30-min washes in PBS. Each ring was then placed in nominally Ca^2+^-free Krebs solution [containing (in mmol/L) 137 NaCl, 2.7 KCl, 8 Na_2_HPO_4_, and 2 KH_2_PO_4_] before being mounted onto the pins of an electromechanical puller (DMT560; Danish Myo Technology A/S, Aarhus, Denmark), calibrated, and aligned. An electromotor slowly moved the pins apart at a rate of 20 μm/s at room temperature in air to apply radial stress on the tissue until breakage. Displacement and force were recorded continuously. A 1 mm segment proximal to the ring was imaged at 10× magnification along with a graticule. The inner and outer diameters of each vessel were measured at four different locations with ImageJ software [National Institutes of Health (NIH), Bethesda, MD]. Averages of these measurements were used to calculate sample thickness. Engineering stress (S) was calculated by normalizing force (F) to the initial stress-free area of the specimen (S = F/2t × l; where t = thickness and l = length of the sample). Engineering strain (λ) was calculated as the ratio of displacement to the initial stress-free diameter. The stress-strain relationship was represented by the equation S = α exp (βλ), where α and β are constants. α and β were determined by non-linear regression for each sample and used to generate stress-strain curves by treating the x-axis as a continuous variable. Incremental elastic modulus (E_inc_) was calculated as the slope of the stress-strain curve at a strain of 0.5, representing elastin-dependent deformation, and at a strain of 1.8, representing the shift to collagen-dependent deformation.

### PWV and Heart Rate Measurements

Mice anesthetized with 2% isoflurane were placed on a heating pad, and their paws were taped to electrocardiogram electrodes. PWV was measured non-invasively as previously described by high-frequency Doppler (Indus Instruments, Webster, TX) at two points along the aorta: one point in the upper thoracic cavity and one point in the lower abdominal cavity (Hartley et al., 1997; Steppan et al., 2019). PWV was calculated as the separation distance divided by the pulse transit time between the two points. Heart rate was calculated by using the distance between two consecutive R waves on the electrocardiogram.

### Non-invasive Blood Pressure Measurements

After slow acclimatization over 2 weeks, animals were restrained in a translucent plastic cone with the tail exposed. Blood pressure was determined by a tail cuff measurement system as described previously (Kent Scientific, Torrington, CT) (Wang et al., 2017; Steppan et al., 2019).

### Histological Staining Techniques

Mouse thoracic aortic rings were cut into 2 mm lengths, formalin fixed, paraffin embedded, sectioned at 6 μm, and mounted onto slides. Hematoxylin and eosin, Masson trichrome, Movat pentachrome, van Gieson, and von Kossa stains were applied in the Department of Pathology Reference Histology laboratory with standard methods. Images were digitally captured with a Laxco microscope at 40× magnification.

### Statistical Analysis

Data are presented as mean ± standard error of the mean (SEM). Sample size (n; number of animals used) is indicated for each reported value. To compare more than one mean, one-way analysis of variance (ANOVA) with Bonferroni *post hoc* analysis was used. For statistical evaluation of multiple comparisons, repeated two-way ANOVA with Bonferroni *post hoc* analysis was used. For curve fitting of the vasoreactivity data, we used a non-linear fit model with a least squares fit. All data analyses were carried out in Prism 8 (GraphPad). Means were considered to be statistically different at *p* < 0.05.

## Results

### Blood Pressure Normalizes After Cessation of AngII Infusion

**FIGURE 1:**
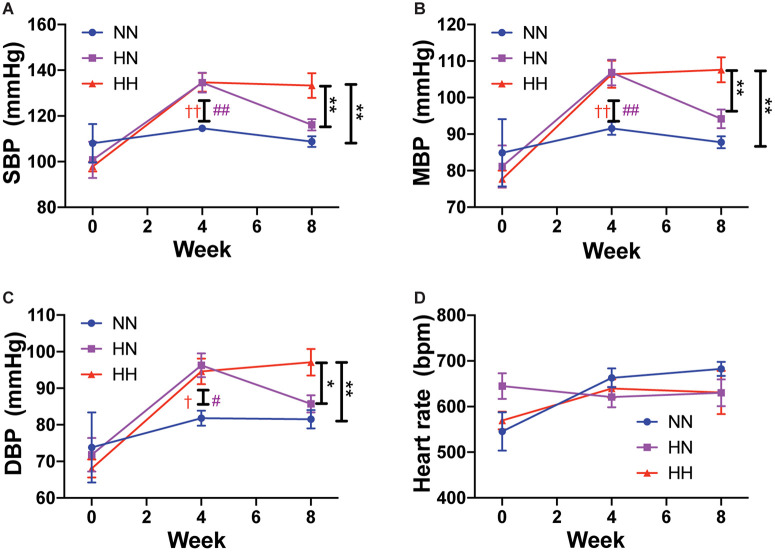
Angiotensin II infusion increases blood pressure but does not affect heart rate. **(A)** Systolic blood pressure (SBP), **(B)** mean blood pressure (MBP), **(C)** diastolic blood pressure (DBP), and **(D)** heart rate in HH, HN, and NN cohorts over the course of the experiment. n = 10 per group. At 8 weeks: ^∗^*p <* 0.05, ^∗∗^*p <* 0.01 by repeated measures 2-way ANOVA with Bonferroni *post hoc* analysis. At 4 weeks: ^#^*p <* 0.05, ^##^*p <* 0.01 for HN vs. NN and ^†^*p <* 0.05, ^††^*p <* 0.01 for HH vs. NN by repeated measures 2-way ANOVA with Bonferroni *post hoc* analysis.

Angiotensin II infusion is a well-known model of experimental hypertension in mice. Consistent with prior studies, systolic BP increased rapidly in the HH and HN groups during the first 4 weeks when compared to NN control BP, which did not change during the course of the experiment. By 4 weeks, systolic BP in the HH and HN groups was significantly higher than that in the NN group (Figure 1A; *p <* 0.01 for HH vs. NN and HN vs. NN). Mean BP (Figure 1B; *p <* 0.01 for HH vs. NN and HN vs. NN) and diastolic BP (Figure 1C; *p <* 0.05 for HH vs. NN and HN vs. NN) were also elevated in the HH and HN groups after 4 weeks of angII infusion. Over the next 4 weeks, systolic, diastolic, and mean BPs of the HN group were restored to levels similar to those of the NN controls. However, systolic (*p <* 0.01 for HH vs. NN and HH vs. HN at 8 weeks), diastolic (*p <* 0.01 for HH vs. NN, *p <* 0.05 for HH vs. HN at 8 weeks), and mean pressures (*p <* 0.01 for HH vs. NN and HH vs. HN at 8 weeks) in the HH group remained significantly higher than those in the NN and HN groups during weeks 4–8. Heart rate was similar in all the cohorts at all time points measured (Figure 1D).

### *In vivo* Vascular Stiffening Persists After Restoration of Normal BP in Previously Hypertensive Animals

**FIGURE 2:**
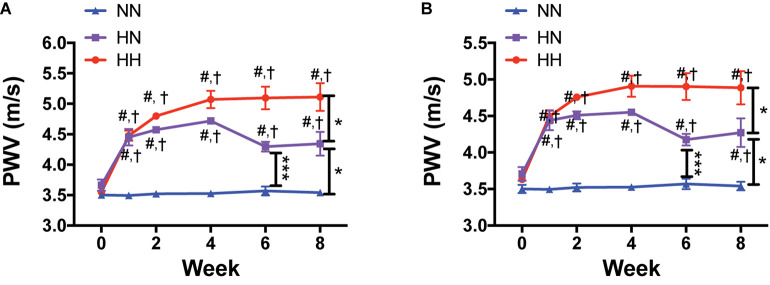
*In vivo* vascular stiffness increases with angII infusion and is partially reversed by restoration of normal blood pressure (BP). **(A)** Longitudinal pulse wave velocity (PWV) as an index of *in vivo* vascular stiffness in HH, HN, and NN cohorts. **(B)** PWV corrected for MAP. *n* = 10 mice per group. ^∗^*p <* 0.05, ^∗∗∗^*p <* 0.001 between indicated groups; ^#^*p <* 0.001 for both HH and HN groups vs. NN at matched time point; ^†^*p <* 0.001 for HH and HN groups vs. corresponding baseline (time 0) by repeated measures 2-way ANOVA with Bonferroni *post hoc* analysis.

We measured PWV as an index of *in vivo* vascular stiffness. Compared to the baseline values measured before angII delivery (week 0), PWV increased rapidly and significantly in the HH and HN groups during the first 4 weeks, but not in NN controls (Figure 2; *p <* 0.001 for both HH and HN groups vs. NN at each time point; *p <* 0.001 for HH and HN groups vs. corresponding baseline; *p <* 0.05 for HH vs. HN and HN vs. NN at 8 weeks). PWV did not differ significantly between the HH and HN groups at 4 weeks (*p* = 0.21) but was significantly higher in both groups than in NN mice. In the HN group, PWV decreased during the 4 weeks after cessation of angII infusion but was only partially restored toward that of NN controls. At the end of the 8-week period, PWV in the HN group was significantly higher than that in the NN controls but significantly lower than that of the HH cohort. PWV was significantly higher in the HH group than in the NN group at both 4 and 8 weeks. A plateau was noted in the HH group, as no further increase in PWV occurred after 4 weeks. PWV remained unchanged in the NN control group over 8 weeks (Figure 2).

### Hypertension Reversal Restores Passive Stiffness of Vascular Matrix in Mice

**FIGURE 3:**
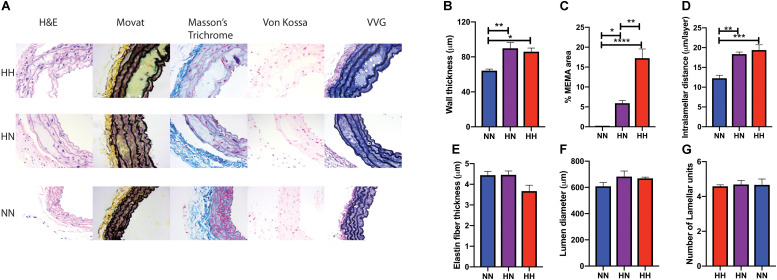
Changes in vascular wall composition and architecture in hypertension reversal. **(A)** Representative histological staining analysis showing hematoxylin and eosin (H&E), collagen/elastin content (Movat staining), the elastic lamella (Masson’s Trichrome staining), calcifications (von Kossa), and elastin (van Gieson) for the three groups (*n* = 5 per cohort). **(B)** Vessel wall thickness, **(C)** percent MEMA area normalized to total vessel wall area, **(D)** intralamellar distance, **(E)** elastin fiber thickness, **(F)** vessel lumen diameter, and **(G)** number of lamellar units in aortas at the end of 8 weeks. *n* = 5 mice per group; ^∗^*p <* 0.05, ^∗∗^*p <* 0.01, ^∗∗∗^*p* < 0.0003, and ^****^p < 0.0001 by ordinary 1-way ANOVA.

*In vivo* vascular stiffness as measured by PWV is the sum total of the contribution of the primary load-bearing elements – the vascular matrix and the VSMCs. Moreover, PWV is significantly affected by BP owing to the viscoelastic nature of blood vessels. Thus, we used *ex vivo* techniques to measure passive stiffness to determine whether the reduction in PWV in the HN cohort is merely due to restoration of normal BP or if it is accompanied by recovery of the vascular wall structure and mechanics. Histological staining analysis (Figure 3A) revealed that restoration of normal BP did not fully reverse changes to the vascular wall architecture and composition wrought by angII infusion. Significant hypertrophy and vascular wall deterioration were observed in the HH mice when compared to NN controls. Alterations included significantly increased wall thickness (Figure 3B; *p <* 0.05, HH vs. NN), intralammelar mucoid extracellular matrix accumulation (MEMA; Figure 3C; *p <* 0.0001, HH vs. NN) (Halushka et al., 2016), and increased intralamellar distance (Figure 3D; *p <* 0.001, HH vs. NN). Average elastin fiber thickness showed a trend toward decrease, but the data did not reach statistical significance (Figure 3E; *p* = 0.08, HH vs. NN). Although the histological changes were more striking in the HH group, significant medial deterioration, including MEMA (*p <* 0.05, HN vs. NN; *p <* 0.001 HH vs. HN), increased wall thickness (*p <* 0.01, HN vs. NN), and increased intralamellar distance (*p <* 0.01, HN vs. NN), were also noted in the HN group, despite cessation of angII infusion. Calcification was not noted in any of the groups (Von Kossa stain; Figure 3A), and lumen diameter and number of lamellar units in the HH and HN groups did not differ significantly from that of the NN controls (Figures 3F,G).

**FIGURE 4:**
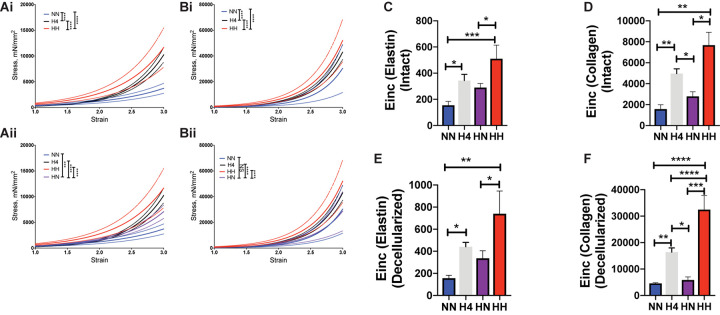
Hypertension causes aortic stiffening that is partially reversed by restoration of blood pressure. Tensile testing of **(Ai,ii)** intact aortas and **(Bi,ii)** decellularized aortas (*n* = 7 mice per group). Data are shown as mean (solid line) ± standard error of the mean (dotted lines of same color). ^∗∗∗^*p <* 0.001, ^****^p < 0.0001 by ordinary 2-way ANOVA with Bonferroni *post hoc* analysis for AngII status and strain. Incremental elastic modulus (E_inc_) of **(C)** intact vessels at low strain of 0.5 representing elastin deformation; **(D)** intact vessels at high strain of 1.8 representing collagen deformation; **(E)** decellularized vessels at low strain of 0.5 representing elastin deformation; **(F)** decellularized vessels at high strain of 1.8 representing collagen deformation. *n* = 7 mice per group, ^∗^*p <* 0.05, ^∗∗^*p <* 0.01, ^∗∗∗^*p <* 0.001, ^****^p < 0.0001 by ordinary 1-way ANOVA with Bonferroni *post hoc* analysis.

Next we examined the relative contributions of the vascular matrix and VSMCs to incomplete reversal of PWV in the HN group by measuring the *ex vivo* passive mechanical properties of the aorta at the end of the study period in all four cohorts. Stiffness of intact and decellularized segments (Figures 4Ai,Bi) increased with angII treatment in the first 4 weeks (intact H4 vs. intact NN, *p <* 0.0001; decellularized H4 vs. decellularized NN, *p <* 0.0001) and increased further in the next 4 weeks of hypertension (intact HH vs. intact H4, *p <* 0.01; decellularized HH vs. decellularized H4, *p <* 0.0001). Restoration of normotension after cessation of angII infusion partially reversed stiffness of intact vessels (Figure 4Aii; intact H4 vs. intact HN, *p <* 0.0001; intact HH vs. intact HN, *p <* 0.0001); however, intact aortic rings from the HN group were significantly stiffer than those from the NN controls (Figure 4Aii; intact HN vs. intact NN, *p* < 0.001).

We next determined if these changes were attributable to elastin-mediated deformation at low strain (= 0.5) or collagen-mediated deformation at high strain (= 1.8) by calculating the incremental elastic modulus (E_inc_) at these respective strain values. In comparison to NN controls, the E_inc_ of intact HH and H4 aorta was significantly higher at low strain (Figure 4C; intact HH vs. NN, *p <* 0.001; HH vs. HN, *p <* 0.05; H4 vs. NN, *p <* 0.05) and at high strain (Figure 4D; intact HH vs. NN, *p <* 0.01; HH vs. HN, *p <* 0.05; H4 vs. NN, *p <* 0.01; H4 vs. HN, *p <* 0.05). In decellularized segments, E_inc_ was higher in HH and H4 cohorts at both low and high strain vs. NN controls (Figures 4E,F, HH vs. NN, *p <* 0.01; H4 vs. NN, *p <* 0.05 at low strain and HH vs. NN, *p <* 0.0001; H4 vs. NN, *p <* 0.01 at high strain). Interestingly stiffening of HH above the H4 cohort was noted only at high strain (HH vs. H4, *p* > 0.05 at low strain; HH vs. H4, *p <* 0.0001 at high strain) and the reversal of stiffness in the HN cohort was statistically significant in the decellularized segments at higher strain (HH vs. HN, *p <* 0.001; H4 vs. HN, *p <* 0.05). Surprisingly, the mechanical properties of decellularized aortic matrices from HN and NN mice were similar (Figures 4Bii,E,F, decellularized HN vs. NN, *p*> 0.05), notwithstanding the structural changes noted in the histological analysis. Together, these findings suggest a significant contribution from the VSMCs to the overall *in vivo* stiffness at the end of the study period despite restoration of the elastic modulus of the decellularized aortic scaffold.

### Vascular Reactivity Is Impaired Despite Restoration of Normal BP in Previously Hypertensive Animals

**FIGURE 5:**
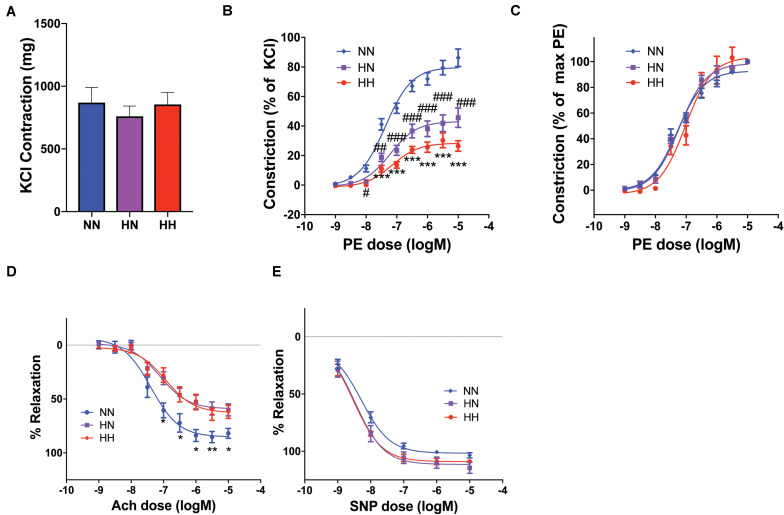
Aortic vasoreactivity is impaired by hypertension despite restoration of blood pressure. **(A)** The maximal contraction response to potassium chloride (KCl) was similar in the three groups (*n* = 8 mice per cohort). **(B)** Contraction response to increasing phenylephrine (PE) concentrations calculated as a percent of maximal KCl-induced contraction was significantly impaired in the HH cohort and modestly better in the HN cohort (*n* = 8 mice per cohort; ^∗∗∗^*p <* 0.001 for HH vs. NN and HH vs. HN; ^#^*p <* 0.05, ^##^*p <* 0.01, ^###^*p <* 0.001 vs. NN by ordinary 2-way ANOVA with Bonferroni *post hoc* analysis). **(C)** EC50 of the cohorts remained similar, as shown by the PE contractile responses normalized to maximal PE contraction for each sample. **(D)** The endothelium-dependent vasodilatory response of PE-pre-constricted vessels to increasing concentrations of acetylcholine (Ach) was impaired in both HN and HH cohorts (*n* = 8 mice per cohort; ^∗^*p <* 0.05, ^∗∗^*p <* 0.01 for NN vs. HN and NN vs. HH by ordinary 2-way ANOVA with Bonferroni *post hoc* analysis). **(E)** Endothelium-independent vasorelaxation in response to increasing concentrations of sodium nitroprusside (SNP) was similar in all groups (*n* = 8 mice per cohort).

Given the significant contribution of VSMCs to the overall vascular modulus identified by PWV measurement and tensile testing, we evaluated the vasoreactivity of the descending thoracic aorta at the end of the study period (8 weeks). The absolute contraction amplitude to KCl was similar in all groups (Figure 5A, HH: 855.8 ± 95.0 mg, HN: 761.1 ± 81.5 mg, NN: 870.0 ± 121.3 mg). The NN group exhibited a robust contractile response to phenylephrine depicted as phenylephrine-induced contraction response normalized to the maximal KCl-induced contraction. The contraction response was markedly attenuated in the HH group compared with that in NN controls (Figure 5B maximum response: HH, 26.4 ± 3.6%; NN, 86.3 ± 6.0%, *p <* 0.0001). However, EC50 did not change (logEC50: HH, −7.15 ± 0.16; NN, −7.38 ± 0.08), as seen by normalizing the contraction with respect to the maximum induced by phenylephrine in each group. The contraction response of the HN group was modestly better than that of the HH group (maximum response: HN, 45.6 ± 6.6%), but it remained significantly attenuated compared with that of the NN controls (HH vs. HN, *p* = 0.6; HH vs. NN, *p* < 0.0001; HN vs. NN, *p* = 0.0004; Figure 5C), again with no changes in EC50 (logEC50: HN, −7.22 ± 0.16) (Spronck et al., 2015, 2018).

Next we investigated the endothelium-dependent vasodilatory response of phenylephrine pre-constricted vessels by applying increasing concentrations of acetylcholine. Because phenylephrine-induced contraction was significantly impaired in the HH and HN groups, it was not possible to achieve an equal magnitude of pre-constriction. Therefore, in these experiments, while the absolute pre-constriction achieved by phenylephrine differed in the HH, HN, and NN groups (HH: 304.5 ± 46.0 mg, HN: 440.7 ± 50.5 mg, NN: 685.6 ± 98.7 mg), it represented equivalent pre-constriction of 90–95% of maximal phenylephrine-induced contraction for each cohort, thus providing sufficient methodologic dynamic range to investigate relaxation responses. The relaxation response was significantly attenuated in both HH and HN groups (HH, 62.0 ± 6.0%; HN, 60.1 ± 5.5%) when compared with that of the NN controls (76.6 ± 6.6%; HH vs. HN, *p* = 0.99; HH vs. NN, *p* = 0.04; HN vs. NN, *p* = 0.02; Figure 5D). The endothelium-independent vasorelaxation response to increasing concentrations of sodium nitroprusside did not differ significantly between the groups (maximum response: HH, 109.0 ± 1.6%; HN, 114.5 ± 4.7%; NN, 103.5 ± 2.1%; *p* > 0.05 for all comparisons; Figure 5E. Together, the vasorelaxation experiments suggest that significant endothelial dysfunction remains in the HN cohort despite the restoration of normal BP upon cessation of angII infusion.

## Discussion

Using the well-established angII infusion model of systemic arterial hypertension (Landmesser et al., 2002; Monassier and El Fertak, 2006), we showed that the induction of hypertension leads to vascular remodeling and overall stiffening of the aorta as a result of both passive vascular matrix stiffening and contributions from VSMCs. We further showed that subsequent restoration of normotension reverses passive stiffness of the aortic matrix, but confers only partial improvement in overall *in vivo* vascular stiffness and phenylephrine-induced vasoconstriction and does not restore endothelial-mediated relaxation response.

The central role of the renin-angiotensin-aldosterone system in the development of essential hypertension is well-known (Forrester et al., 2018). Overactivity of this system increases angII generation, which leads to essential hypertension. Administration of angII in rodent models revealed that disturbances in NADPH oxidase-dependent reactive oxygen species, recruitment of T-cells, development of atherosclerosis, and remodeling of the extracellular matrix all contribute to the effects of angII, eventually leading to vascular stiffening (Landmesser et al., 2002; Wang and Fitch, 2004; Zhao et al., 2004; Guzik et al., 2007). Arterial stiffness is an important indicator of cardiovascular health and a predictor of morbidity and mortality independent of other risk factors, including hypertension (Amar et al., 2001; Sato et al., 2005; Sutton-Tyrrell et al., 2005). Vascular stiffening and hypertension go hand-in-glove, with stiffening preceding the onset of hypertension in aging and diet-induced obesity (Weisbrod et al., 2013; Sun, 2015), and hypertension being causal in arterial stiffening that accompanies essential hypertension. Although hypertensive pressure itself can manifest as augmented PWV due to the non-linearly viscoelastic nature of the blood vessels, the remodeling and stiffening of vessels in essential hypertension is incontrovertible. At the cellular/molecular level, the composition and mechanical properties of the vascular matrix are an important determinant of overall vascular stiffness. Prior studies have clearly shown that antihypertensive treatment has the additional benefit of ameliorating vascular stiffening in patients with hypertension (Ong et al., 2011; Hayoz et al., 2012; Bramlage et al., 2013; Jia et al., 2018). Medications that have vasodilatory properties, such as angiotensin converting enzyme inhibitors or calcium channel blockers, are efficacious in reducing arterial stiffness, particularly when used in combination with a diuretic (Safar and Jankowski, 2010; Cameron et al., 2013). Therefore, lowering blood pressure has been described as the most important mechanism by which antihypertensive drugs improve overall vascular stiffness (Safar and Jankowski, 2010). Furthermore, it has been observed previously in hypertensive patients that stiffness of the radial artery vessel wall is not increased despite being hypertrophied (Liao et al., 2016). In agreement with these prior studies, our experiments show that the macroscopic tensile properties of the vascular matrix are indeed restored to levels similar to those of normotensive controls despite the sustained hypertrophy and deterioration of the vascular wall structure and composition in the HN group after the restoration of normal BP.

Importantly, PWV, an index of *in vivo* stiffness, remains elevated despite the benefits observed with respect to both BP and matrix mechanics after cessation of angII infusion in the HN group. This finding points to causes other than matrix remodeling as being responsible for augmented PWV in the HN group. Recent studies have revealed a central role for the VSMCs themselves in modulating *in vivo* arterial stiffness – specifically, VSMC tone (active component), intrinsic stiffness of VSMCs themselves, and/or changes in the VSMC/matrix interactions (number and strength of focal adhesions) all contribute to overall *in vivo* vascular stiffness (Sehgel et al., 2015; Steppan et al., 2017, 2019; Lacolley et al., 2018). When considered together, our findings support this new paradigm that the matrix (passive) and the VSMCs (active) are equal shareholders in the sustained increase in PWV noted in the HN cohort.

Our vasoreactivity studies showed that while maximal contraction in response to KCl is similar in HH and NN mice, agonist-induced contraction is impaired in the VSMCs of the HH cohort. Moreover, this impairment persists in the HN cohort despite restoration of normal BP, and in the context of similar maximal contraction in response to KCl. Specifically, EC50 was not affected, but rather the magnitude of contraction was compromised. Similarly, endothelial-mediated relaxation was attenuated, indicating that endothelial dysfunction is also present in the HN cohort. Maximal contraction to phenylephrine was impaired in the HH group and was still significantly reduced in the HN group. Thus, in terms of the active components of arterial wall stiffness, this blunting of agonist-induced contraction equates with reduced contribution to active stiffness. Conversely, the impaired endothelium-dependent relaxation in HN and HH groups would contribute to an increase in the active tone and thus, overall stiffness. *In vivo* basal tone would then depend on the balance between the two. In this context, although endothelial cells themselves do not contribute to mechanical load-bearing, they contribute significantly to overall *in vivo* stiffness (i.e., PWV) by regulating *in vivo* active VSMC tone, function, and phenotype. Therefore, we postulate that *in vivo* endothelial dysfunction and VSMC dysregulation together underlie the incomplete reversal of PWV in the HN group, despite restoration of BP. Specifically, if agonist-induced constriction were the predominant mechanism, basal tone would be lower, and would contribute to the partial recovery of PWV in the HN cohort when compared with the HH group. However, if endothelial dysfunction and the resultant increase in basal tone were the dominant cellular mechanism, VSMCs would contribute to the persistent elevation of PWV in the HN cohort noted in comparison to the NN cohort. The relative contributions of each of these mechanisms, and thus basal tone, remain unstudied. Another unanswered question is the timing of these changes in relation to endothelial and SMC function, as we did not evaluate vasoreactivity in the H4 cohort.

Finally, it is likely that the hypertrophy, composition/structural changes in the HN group evidenced by MEMA, and increased intralamellar distance promote outside-in signaling arising from the localized biomechanical changes. This shift would not only significantly influence the VSMC dysfunction via dedifferentiation or phenotype switching, cause increased VSMC stiffness, and result in the formation of larger cell-matrix adhesions in VSMCs (Sehgel et al., 2015; Hytonen and Wehrle-Haller, 2016), but also promote the endothelial dysfunction that persists in the HN group. Continued endothelial dysfunction would further exacerbate VSMC dedifferentiation and dysfunction. Therefore simply lowering BP without an additional mechanism targeting these molecular and cellular changes noted in the vascular media is not sufficient to fully reverse overall vascular stiffness. This conclusion is corroborated by, among others, a study from Liao et al. (2016), who have shown that reversing hypertension in patients with primary aldosteronism by removing the culprit adenoma improves arterial stiffness to a certain extent early after removal, but fails to fully reestablish vascular compliance. In our study, we noted significant accumulation of MEMA in the HN cohort that did not occur in NN controls, with a further striking increase of MEMA in the HH cohort. This finding suggests that by 4 weeks of hypertension, animals accumulate significant levels of MEMA that do not dissipate by 4 weeks of normotension. MEMA is composed of glycosaminoglycans, a class of structural molecules that accumulate in areas of vasculature that are susceptible to disease initiation and progression (Laurent et al., 1994). This, in association with increased intralamellar distance, is indicative of matrix protein accumulation in the aorta of HN animals. Production of matrix proteins such as collagen and fibronectin, as well as matrix crosslinking enzymes such as amine oxidases (LOX/LOXL2) and transglutaminase 2, is augmented during vascular remodeling (Santhanam et al., 2010; Jung et al., 2013; Steppan et al., 2014, 2017, 2019; Liao et al., 2016). Together, these proteins catalyze the incorporation of matrix proteins via highly stable covalent bonds in the vascular matrix that are not readily degraded *in vivo*. Thus, because of the relatively long half-life of matrix crosslinked collagen and other structural glycoproteins present in the MEMA *in vivo*, it is possible that the time course of recovery is longer than the 4-week recovery period examined here. Therefore, longer outcome periods should be examined in future studies.

Limitations of our studies include the use of an animal model of hypertension that by its nature can only partially recapitulate the pathology of human hypertension. PWV measurement was not experimentally corrected to account for changes in BP in the HH group. Measurements of tensile properties and vascular reactivity were carried out *ex vivo*, and the vasodilatory responses to both acetylcholine and sodium nitroprusside were evaluated after a fixed response to phenylephrine at different magnitudes of pre-constriction. Because we did not measure basal tone, it is unclear whether VSMC contraction contributes to the decrease in PWV in the HN cohort relative to the HH cohort or to the elevated PWV in the HN cohort relative to the NN cohort. Additionally, aortic rings were placed in nominally calcium-free solution prior to tensile testing, meaning that these measurements were not entirely passive in nature. Finally, vascular reactivity and histochemical analyses of mice after 4 weeks of hypertension in the H4 cohort are needed to determine the temporal trajectory of structural and functional vascular changes.

## Conclusion

In conclusion, we showed that restoration of normal BP in hypertensive mice results in a partial recovery of overall *in vivo* stiffness (PWV) in the timeframe studied. We postulate that the reversal arises in part from the restoration of BP, owing to the viscoelastic nature of blood vessels, and in part from the recovery of matrix mechanics. The stiffness that is irreversible results from endothelial dysfunction and molecular changes to the vascular matrix, which together contribute to VSMC dysregulation. The sustained cellular changes are a major contributor to the overall *in vivo* vascular stiffness in essential hypertension. Together, these findings suggest that a second or later vascular insult could potentially elicit an accelerated and exaggerated response, but this possibility remains to be tested.

## Data Availability Statement

The datasets analyzed for this study are available on request to the corresponding author.

## Ethics Statement

The animal study was reviewed and approved by the Johns Hopkins University IACUC.

## Author Contributions

JS performed the experiments, analyzed the data, and wrote and revised the manuscript. SJ, WS, HW, SK, and RN performed the experiments and analyzed the data. DN edited the manuscript. LS designed the study, provided the funding, analyzed the data, and edited the manuscript. All authors contributed to the article and approved the submitted version.

## Conflict of Interest

The authors declare that the research was conducted in the absence of any commercial or financial relationships that could be construed as a potential conflict of interest.
